# Metabolic Profiling of Rumen Fluid and Milk in Lactating Dairy Cattle Influenced by Subclinical Ketosis Using Proton Nuclear Magnetic Resonance Spectroscopy

**DOI:** 10.3390/ani11092526

**Published:** 2021-08-27

**Authors:** Jun-Sik Eom, Hyun-Sang Kim, Shin-Ja Lee, You-Young Choi, Seong-Uk Jo, Jaemin Kim, Sang-Suk Lee, Eun-Tae Kim, Sung-Sill Lee

**Affiliations:** 1 Division of Applied Life Science (BK21), Gyeongsang National University, Jinju 52828, Korea; skyandstar07@naver.com (J.-S.E.); 2437401@naver.com (H.-S.K.); dudolboy301@naver.com (Y.-Y.C.); jsu9412@naver.com (S.-U.J.); jmkim85@gnu.ac.kr (J.K.); 2 Institute of Agriculture and Life Science, Gyeongsang National University, Jinju 52828, Korea; tlswk1000@hanmail.net; 3 University-Centered Labs, Gyeongsang National University, Jinju 52828, Korea; 4 Ruminant Nutrition and Anaerobe Laboratory, Department of Animal Science and Technology, Sunchon National University, Suncheon 57922, Korea; rumen@sunchon.ac.kr; 5 Dairy Science Division, National Institute of Animal Science, Rural Development Administration, Cheonan 31000, Korea; etkim77@korea.kr

**Keywords:** lactating dairy cattle, metabolites, milk, rumen fluid, subclinical ketosis, ^1^H-NMR spectroscopy

## Abstract

**Simple Summary:**

Ketosis metabolic research is extremely rare in Korea. This study aimed to compare the rumen fluid and milk metabolites between healthy and subclinical ketosis-diagnosed lactating dairy cattle. Six Holstein cows were allocated into two groups based on whether they fit the criteria for subclinical ketosis, and their rumen fluid and milk samples were collected from the stomach tube and pipeline milking system. Rumen fluid and milk samples metabolites were analyzed using proton nuclear magnetic resonance spectroscopy. They were identified and quantified using the Chenomx NMR Suite 8.4 software and statistical analysis was performed using Metaboanalyst 5.0. In rumen fluid, ruminant energy source metabolites (acetate, glucose, and propionate) were significantly higher in the healthy group, whereas in milk, ketone body metabolites (3-hydroxybutyrate and acetoacetate) were significantly higher in the subclinical ketosis-diagnosed group. This report will serve as a reference guide for future studies on ketosis metabolomics in Korea.

**Abstract:**

Ketosis metabolic research on lactating dairy cattle has been conducted worldwide; however, there have been very few Korean studies. Biofluids from lactating dairy cattle are necessary to study ketosis metabolic diseases. Six Holstein cows were divided into two groups (healthy (CON) and subclinical ketosis diagnosed (SCK)). Rumen fluid and milk samples were collected using a stomach tube and a pipeline milking system, respectively. Metabolites were determined using proton nuclear magnetic resonance (NMR) spectroscopy and they were identified and quantified using the Chenomx NMR Suite 8.4 software and Metaboanalyst 5.0. In the rumen fluid of the SCK group, butyrate, sucrose, 3-hydroxybutyrate, maltose, and valerate levels were significantly higher than in the CON group, which showed higher levels of *N*,*N*-dimethylformamide, acetate, glucose, and propionate were significantly higher. Milk from the SCK group showed higher levels of maleate, 3-hydroxybutyrate, acetoacetate, galactonate, and 3-hydroxykynurenine than that from the CON group, which showed higher levels of galactitol, 1,3-dihydroxyacetone, γ-glutamylphenylalanine, 5-aminolevulinate, acetate, and methylamine. Some metabolites are associated with ketosis diseases and the quality of rumen fluid and milk. This report will serve as a future reference guide for ketosis metabolomics studies in Korea.

## 1. Introduction

Ketosis is a metabolic disease in lactating dairy cattle characterized by high concentrations of ketone bodies (3-hydroxybutyrate [BHBA], acetoacetate, and acetone) metabolites in their blood (plasma and serum), milk, and urine [1]. Ketosis is classified into two groups; subclinical ketosis (SCK) (concentration of BHBA in serum are between 1.2 to 1.4 mM/L) and clinical ketosis (concentration of BHBA in serum are between 2.6 to 3.0 mM/L) [2,3]. The causes of ketosis in lactating dairy cattle are early lactation, high energy requirements for milk production combined with comparatively low feed intake [4,5], and extreme reduction in feed intake during peripartum [6]. The negative effects of ketosis may include decreased milk production, reduced reproduction, higher risk of lameness, mastitis, metritis, retained placenta, and increased culling ratio [7,8,9,10]. Therefore, more studies on ketosis in lactating dairy cattle are required.

Numerous studies on lactating dairy cattle ketosis disease using various metabolite detection techniques such as nuclear magnetic resonance (NMR) spectroscopy, gas chromatography-mass spectrometry (GC-MS), and liquid chromatography-mass spectrometry (LC-MS) have focused on the metabolite changes in their biofluids (rumen fluid, serum, plasma, milk, urine, and feces).

In studies on rumen fluid, GC has been used to compare the bacterial community through volatile fatty acid (VFA) production [11] and acetone through isopropanol concentrations [12,13]. However, research using rumen fluid has not been performed yet. In studies on milk, NMR spectroscopy has been used to compare glycerophosphocholine (GPC) using the phosphocholine ratio [14], whereas GC-MS was used for other metabolites associated with amino acids and carboxylic acid [15]. Furthermore, metabolites in lactating dairy cattle milk were studied to compare and identify biomarkers for lactating dairy cattle ketosis disease [16,17,18]. Xu et al. [16] suggested glycine in milk as a potential biomarker for ketosis; furthermore, Xu et al. [18] reported that glycine, choline, and carnitine play important roles in ketosis.

In Korea, the Holstein species constitute approximately 99% of the lactating dairy population and are the most important livestock breed in the dairy industry [19]. Many studies have been conducted on the improvement of milk production, composition, and associated diseases in Holstein cows. Above all, research on ketosis has focused on ketone body metabolite concentrations in blood, milk, and urine [20,21,22]. Recently, metabolomics studies have been conducted by comparing biofluid metabolites from Korean native cattle (Hanwoo; *Bos taurus coreanae*), and lactating dairy cattle (Holstein species) using proton nuclear magnetic resonance (^1^H-NMR) spectroscopy [23,24,25]. However, owing to the low number of studies using ^1^H-NMR spectroscopy on lactating dairy cattle biofluid metabolites associated with ketosis metabolic diseases, more studies are required.

This study showed that the metabolic profiles of Holstein cows based on rumen fluid and milk might differ between the healthy (CON) and SCK groups. Based on the rumen fluid and milk samples, this study aimed to elucidate the metabolic profiles of Holstein cows by using ^1^H-NMR spectroscopy and to compare the CON and SCK Holstein groups. Moreover, studies on metabolites using ^1^H-NMR spectroscopy are insufficient in Korea, this study will serve as a reference guide for future ruminant’s ketosis metabolomics studies.

## 2. Materials and Methods

### 2.1. Animals and Sampling

Three CON Holstein cows (39.27 ± 3.39-months-old; body weight, 554.33 ± 19.30 kg; parity, 1.00 ± 0.00; milk yield, 30.30 ± 5.75 kg; blood BHBA concentration, 0.63 ± 0.12 mM) and three SCK Holstein cows (56.91 ± 24.89-month-old; body weight, 563.33 ± 65.19 kg; parity, 1.67 ± 1.15; milk yield, 25.33 ± 2.04 kg; blood BHBA concentration, 1.33 ± 0.78 mM) were used in this study.

The experiment lasted 14 days, with first 4 days as the diet adaptation period. The blood BHBA concentration was measured after milking in the morning and it was conducted for a total of 9 days during the monitoring period. The range of blood BHBA concentrations for the SCK group was established as 1.0 to 1.4 mM, whereas for the CON group, it ranged between 0.5 to 0.7 mM [2,26,27,28]. The blood BHBA concentration was determined using a portable ketone test meter (FreeStyle Optium Neo H-Ketone Meter; Abbot Diabetes Care Ltd., Witney, Oxon, UK) and the respective strips (Precision Extra Ketone Test Strips) by following the instructions provided by the manufacturer.

Rumen fluid and milk samples were collected on the last day of the experiment. All the animals were fed total mixed ration (TMR). The amount of TMR consumed by cows of the CON group depended on their voluntary intake; however, the cows of the SCK group had a restricted feed intake in the stanchion barn (30 kg/day; approximately 10 kg per animal). Table 1 shows the chemical composition results of TMR. The contents of dry matter (method No. 934.01), crude protein (method No. 976.05), calcium (method No. 927.02), and phosphorus (method No. 3964.06) in TMR was assayed as described by Association of Official Analytical Communities methods [29]. The contents of neutral detergent fiber and acid detergent fiber in TMR were assayed as described by Van Soest et al. [30].

Rumen fluid was collected from the Holstein cows using the stomach tube 3 to 4 h after their morning feed. The first rumen fluid was not sampled due to saliva and blood contamination. All rumen fluid was collected using a conical tube (30 mL each). Subsequently, rumen fluid samples were centrifuged at 806× *g* for 15 min to remove feed particles and the supernatant was stored at −80 °C until analyzed for metabolites using ^1^H-NMR spectroscopy [23]. Milk samples were collected by using a pipeline milking system and then transferred to a conical tube (30 mL). Subsequently, milk samples were stored at −80 °C until analyzing for metabolites using ^1^H-NMR spectroscopy [23].

### 2.2. Prepared Proton Nuclear Magnetic Resonance Spectroscopy Analyses

The rumen fluid samples were recentrifuged at 12,902× *g* for 10 min and the supernatant was collected 300 μL. Standard buffer solution (2,2,3,3-d(4)-3-(trimethylsilyl) propionic acid [TSP] sodium salt) was added to 300 μL of supernatant in deuterium oxide (D_2_O) solvent / standard buffer solution (300 μL). The supernatants (600 μL) were transferred to 5 mm NMR tubes for NMR analysis [31,32]. The collected milk samples were centrifuged at 4000× *g* for 15 min to remove the lipid layer in the supernatant. Thereafter, the mixture of milk (250 μL) and D_2_O (300 μL) were transferred to 5 mm NMR tubes for NMR spectroscopy analysis [32,33].

Proton nuclear magnetic resonance spectroscopy spectra of rumen fluid and milk samples were obtained on an SPE-800 MHz NMR-MS spectrometer (Bruker BioSpin AG, Fällanden, Switzerland) at 64 K using a 5 mm triple-resonance inverse cryoprobe with Z-gradients (Bruker BioSpin CO., Billerica, MA, USA). The pulse sequence used for the rumen fluid and milk were per-saturation pulse sequence collecting 64,000 data points with 128 transients, a spectral width of 16,025.641 Hz, a relaxation delay of 4.0 s and an acquisition time of 2.0 s.

### 2.3. Metabolites Identification, Quantification, and Statistical Analyses

The metabolites qualitative and quantification were carried out by import the analyzed spectral data into the Chenomx NMR suite 8.4 software (ChenomxInc, Edmonton, AB, Canada). The baseline and phase were matched for comparison between samples using the Chenomx processor. The spectral width was 10 ppm and was referenced to the TSP signal at 0 ppm. Metabolite qualitative and quantitative analyses were performed by using LMD (livestock metabolites database; [http://www.lmdb.ca, accessed on 12 March 2021]), BMD (bovine metabolite database; [http://www.bmdb.ca, accessed on 12 March 2021]), and the Chenomx profiler.

Statistical analyses of the metabolites data were using the Metaboanalyst version 5.0 (http://www.metaboanalyst.ca, accessed on 16 March 2021). The resulting data were normalization selected methods were, as follows, raw wise normalization: normalization to constant sum; data transformation: log normalization; data scaling: pareto scaling. Univariate Student’s *t*-test was used to quantify the difference between metabolite profiles of the rumen fluid and milk. Principal component analysis (PCA) and partial least square-discriminant analysis (PLS-DA) were used as multivariate data analysis techniques to generate a classification model and provide quantitative information for discriminating the rumen fluid and milk sample metabolites. The different CON and SCK group’s rumen fluid and milk metabolites were determined on the basis of a statistically significant threshold of variable importance in projection (VIP) scores. Metabolites with VIP scores higher than were obtained 1.5 were obtained PLS-DA model. Metabolic pathway analysis was performed using a *Bos taurus* pathway library. Metabolic pathways were significantly different; rumen fluid and milk metabolites of the other studied animals were statistically analyzed by Metaboanalyst version 5.0 for metabolic pathways analysis, which were based on database sources by KEGG (kyoto encyclopedia of genes and genomes; [http://www.kegg.com, accessed on 18 March 2021]).

## 3. Results

### 3.1. Multivariate Data Analysis

**Figure 1:**
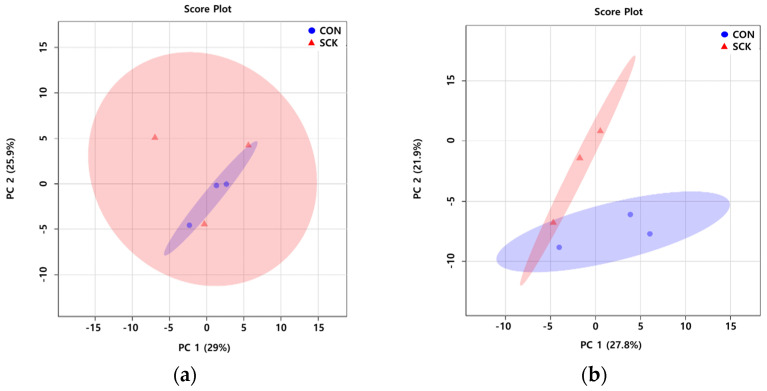
Principal components analysis (PCA) score plot based on rumen fluid (**a**) and milk (**b**) metabolites data in healthy (CON) and subclinical ketosis (SCK) groups by proton nuclear magnetic resonance analysis (*n* = 3). On the score plot, each point represents an individual sample, with the blue circle representing the CON, and the red triangle representing the SCK group. The abscissa and represent the variance associated with PC 1 and 2, respectively.

To characterize the variations in the rumen fluid and milk metabolic profiles of the CON and SCK groups, PCA and PLS-DA were conducted. In the rumen fluid PCA score plots (Figure 1a), CON and SCK groups were not separated; PC 1 and PC 2 accounted for 29 and 25.9% of the variation, respectively. The milk PCA score plots (Figure 1b) in the CON and SCK groups were separated; PC 1 and PC 2 accounted for 27.8 and 21.9% of the variation, respectively.

**Figure 2:**
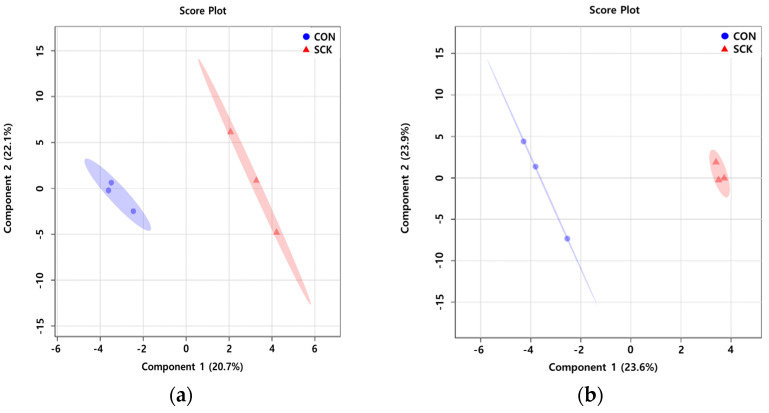
Partial least square-discriminant analysis score plot of rumen fluid (**a**) and milk (**b**) with healthy (CON) and subclinical ketosis (SCK) groups by proton nuclear magnetic resonance analysis (*n* = 3). The shaded ellipses represent the 95% confidence interval estimated from the score. On the score plot, each represents an individual sample, with the blue circle representing the CON, and the red triangle representing the SCK group. The abscissa and ordinate represent the variance associated with component 1 and 2, respectively.

The rumen fluid PLS-DA score plots (Figure 2a) for the CON and SCK groups were clearly separated; components 1 and 2 accounted for 20.7 and 22.1%, respectively. The milk PLS-DA score plots (Figure 2b) for the CON and SCK groups were clearly separated; components 1 and 2 accounted for 23.6 and 23.9%, respectively. These results indicate differences in the classes and concentrations of rumen fluid and milk metabolites identified in the CON and SCK groups.

### 3.2. Detection and Quantification of Rumen Fluid and Milk Metabolites

The results in Table 2, Supplementary Tables S1–S4, and Figures S1 and S2 reveal the metabolites detected and quantified in rumen fluid and milk from the CON and SCK groups. In the CON group, out of the 145 metabolites detected in the rumen fluid, 48 were quantified and classified into 13 chemical classes. In the SCK group, out of 171 metabolites detected in the rumen fluid, 45 were quantified and classified into 13 chemical classes.

In the CON group, out of 163 metabolites detected in the milk, 69 were quantified and classified into 14 chemical classes. In the SCK group, out of 162 metabolites detected in the milk, 84 were quantified and classified into 13 chemical classes.

### 3.3. Differences in Rumen Fluid and Milk Metabolites

Table 3 shows the significance (*p* < 0.05) and trends (0.05 ≤ *p* < 0.1) of different metabolites in the rumen fluid and milk of the CON and SCK groups. In the SCK group, rumen fluid showed significantly higher (*p* < 0.05) butyrate, sucrose, BHBA, maltose, and valerate levels than that in the CON group, and methylamine, methionine, and isopropanol showed a tendency to be higher (0.05 ≤ *p* < 0.1). In contrast, *N*-*N*-dimethylformamide, acetate, glucose, and propionate were significantly (*p* < 0.05) higher in the rumen fluid from the CON group than in that from the SCK group. In the milk from the SCK group, maleate, BHBA, acetoacetate, galactonate, and 3-hydroxykynurenine were significantly (*p* < 0.05) higher, and acetone, guanidoacetate, 2-oxoisocaproate, xanthine, and trehalose showed a tendency to be higher (0.05 ≤ *p* < 0.1) compared to that in the CON group. In contrast, galactitol, 1,3-dihydroxyacetone, γ-glutamylphenylalanine, 5-aminolevulinate, acetate, and methylamine were significantly (*p* < 0.05) higher, and riboflavin and choline levels showed a tendency to be higher (0.05 ≤ *p* < 0.1) in the milk from the CON group than in that from the SCK group.

**Figure 3:**
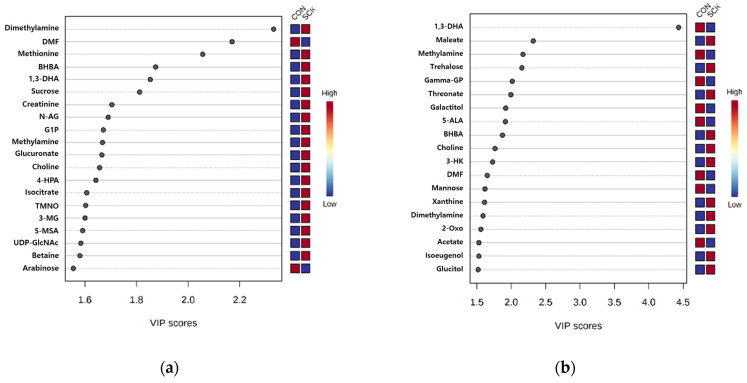
Variable importance in projection (VIP) scores of rumen fluid (**a**) and milk (**b**) metabolites in healthy (CON) and subclinical ketosis (SCK) groups by proton nuclear magnetic resonance analysis (*n* = 3). The selected metabolites were those with VIP score > 1.5. Heat map with red or blue squares on the right indicates high and low abundance ratio, respectively, of the corresponding rumen fluid and milk metabolites in CON and SCK groups. The VIP score was based on the partial least square-discriminant analysis model. Rumen fluid metabolites VIP score value: dimethylamine, 2.3314; DMF, 2.1702; methionine, 2.0563; BHBA, 1.8739; 1,3-DHA, 1.8533; sucrose, 1.8124; creatinine, 1.7047; N-AG, 1.6904; G1P, 1.6713; methylamine, 1.6682; glucuronate, 1.6658; choline, 1.657; 4-HPA, 1.6428; isocitrate, 1.6071; TMNO, 1.6032, 3-MG, 1.6007, 5-MSA, 1.5914; UDP-GlcNAc, 1.5841, betaine, 1.5809, arabinose, 1.5551. Milk metabolites VIP score value: 1,3-DHA, 4.434; maleate, 2.3194; methylamine, 2.1698; trehalose, 2.1553; gamma-GP, 2.0138; threonate, 1.9941; galactitol, 1.9191; 5-ALA, 1.9142; BHBA, 1.872; choline, 1.7636; 3-HK, 1.7289; DMF, 1.6514; mannose, 1.6175; xanthine, 1.6114; dimethylamine, 1.5897; 2-Oxo, 1.5578, acetate, 1.5286; isoeugenol, 1.5282, glucitol, 1.5175. Metabolites abbreviation: DMF, *N*,*N*-dimethylformamide; BHBA, 3-hydroxybutyrate; 1,3-DHA, 1,3-dihydroxyacetone; *N*-AG, *N*-acetylglutamine; G1P, Glucose-1-phosphate; 4-HPA, 4-hydroxyphenylacetate; TMNO, trimethylamine *N*-oxide; 3-MG, 3-methylglutarate; 5-MSA, 5-methoxysalicylate; UDP-GlcNAc, UDP-*N*-acetylglucosamine; Gamma-GP, γ-glutamylphenylalanine; 5-ALA, 5-aminolevulinate; 3-HK, 3-hydroxykynurenine; 2-Oxo, 2-oxoisocaproate.

As shown in Figure 3, evaluation of VIP scores obtained from PLS-DA provided 20 and 19 significantly different metabolites (VIP score > 1.5) between the CON and SCK groups of rumen fluid and milk, respectively. In the rumen fluid, dimethylamine, methionine, and BHBA had the highest VIP scores in the SCK group compared to that in the CON group (Figure 3a). In contrast, *N*,*N*-dimethylformamide and arabinose had the highest VIP scores in the CON group, compared to the SCK group. In milk, maleate, trehalose, and threonate had the highest VIP scores in the SCK group compared to the CON group. In contrast, 1,3-dihydroxyacetone, methylamine, and γ-glutamylphenylalanine had the highest VIP scores in the CON group compared to the SCK group (Figure 3b).

### 3.4. Metabolic Pathway Analysis

**Figure 4:**
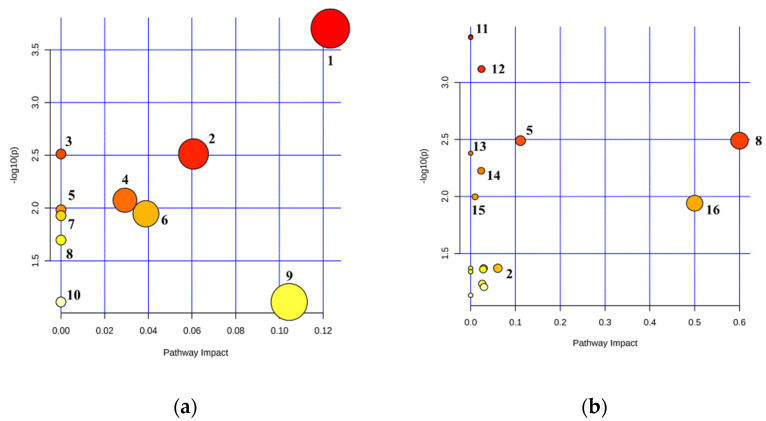
Metabolic pathway mapping was significantly different between rumen fluid (**a**) and milk (**b**) compared in healthy and subclinical ketosis groups. The pathway impact analysis was performed using the Metaboanalyst 5.0 program. The results are presented graphically as a bubble plot. The darker color and larger size represent higher *p*-value from enrichment analysis and greater impact from pathway topology analysis, respectively. Metabolic pathway name: 1, starch and sucrose metabolism; 2, pyruvate metabolism; 3, glyoxylate and dicarboxylate metabolism; 4, glycolysis and gluconeogenesis; 5, butanoate metabolism; 6, galactose metabolism; 7, propanoate metabolism; 8, synthesis and degradation of ketone bodies; 9, cysteine and methionine metabolism; 10, aminoacyl-tRNA biosynthesis; 11, glycerolipid metabolism; 12, glycine, serine and threonine metabolism; 13, tyrosine metabolism; 14, arginine and proline metabolism; 15, valine, leucine and isoleucine degradation; 16, riboflavin metabolism.

Based on rumen fluid metabolism, including that of starch and sucrose metabolism; pyruvate metabolism; glyoxylate and dicarboxylate metabolism; glycolysis and gluconeogenesis; butanoate metabolism; galactose metabolism; propanoate metabolism; and synthesis and degradation of ketone bodies; eight metabolic pathways significantly differed (*p* < 0.05) between the CON and SCK groups. The two metabolic pathways tended to differ (0.05 ≤ *p* < 0.1) between the CON and SCK groups in rumen fluid, including cysteine and methionine metabolism, and aminoacyl-tRNA biosynthesis (Table 4 and Figure 4a).

In milk, 14 metabolic pathways significantly differed (*p* < 0.05) between the CON and SCK groups. These included those involved in galactose metabolism, glycerolipid metabolism; glycine, serine, and threonine metabolism; synthesis and degradation of ketone bodies; butanoate metabolism; tyrosine metabolism; arginine and proline metabolism; valine, leucine, and isoleucine degradation; riboflavin metabolism; pyruvate metabolism; glycolysis and gluconeogenesis; glyoxylate and dicarboxylate metabolism; porphyrin and chlorophyll metabolism; and valine, leucine, and isoleucine biosynthesis. Three metabolic pathways between the CON and SCK groups tended to differ (0.05 ≤ *p* < 0.1), in milk, including glycerophospholipid metabolism, purine metabolism, and starch and sucrose metabolism (Table 4 and Figure 4b).

## 4. Discussion

In this investigation, we have used a relatively small number of Holstein cows; thus, our study may have fewer experimental animals than what is necessary for adequate statistical power analysis [8]. Therefore, the small scale of this experiment may have resulted in fewer significant findings or weaker external validity in comparison to large-scale studies [8]. However, in this study, simultaneous metabolic profiling of rumen fluid and milk samples has been conducted, and this can serve as a guide for future research of SCK in lactating dairy cattle.

Volatile fatty acids, such as acetate, propionate, butyrate, and valerate, accounted for approximately 70% of the ruminant energy sources [34]. However, the excessively high concentrations of butyrate and valerate in the rumen of lactating dairy cattle with ketosis are a concern. Approximately 26–33% butyrate and 18–24% valerate, which were absorbed by the rumen papillae, were converted into BHBA [35]. Feeds with a high concentration of butyrate were considered ketogenic precursors [36]. Robertson and Thin [37] and Thin and Robertson [38] reported that ketotic lactating dairy cattle are associated with high BHBA concentrations in the rumen fluid. In the present study, butyrate and BHBA levels were significantly higher in the SCK group than in the CON group (*p* < 0.05). Rumen in isopropanol absorbed the ketosis signals from the central nervous system [39]. Sato and Shiogama [12] reported that ketosis is related to the presence of acetone and isopropanol in the rumen fluid. In the present study, the isopropanol tended to be higher in the SCK group (0.05 ≤ *p* < 0.1) than in the CON group.

Mellado et al. [40] reported that parturition under heat stress (HS) results in the development of SCK in lactating dairy cattle. In addition, *Acetobacter* in the rumen produces acetate by oxidizing sugars, and its abundance is reduced under HS [41]. The acetate in rumen fluid is absorbed by blood vessels for synthesizing fatty acids in mammary epithelial cells (associated with milk fat) [42]. Succinate is a precursor of propionate, which is associated with gluconeogenesis in ruminants [43]. Clemmons et al. [44] reported that the succinate levels were higher in a low-residual feed intake steer group than in a high-residual feed intake steer group. Propionate is absorbed through the rumen epithelium and used in the liver for gluconeogenesis. Besides, increasing milk production in lactating dairy cattle [45]. Ketosis-induced lactation in dairy cattle has a negative effect on milk composition and milk production [7,9]. In the present study, acetate and propionate levels in the SCK group were significantly lower (*p* < 0.05) than those in the CON group, and succinate was quantified only in the CON group. Glucose in the rumen fluid is an important nutrient required for milk synthesis and is rapidly converted into VFAs [4]. In the present study, the glucose levels in the CON group were significantly higher (*p* < 0.05) than that in the SCK group. Moreover, glycolysis and gluconeogenesis metabolic pathways differed (*p* < 0.05). In addition, the propionate and glucose concentrations were significantly higher in the CON group than in the SCK group. As ketosis research using metabolites from rumen fluid is limited, these results will aid in future studies.

Ketone body metabolites in milk are used to diagnose ketosis [1]. McArt et al. [46] reported that SCK in lactating dairy cattle increased the BHBA concentrations in serum to 100 μM/L and decreased milk yield by 500 mL/day for one month. Milk BHBA concentrations were increased, and high proportions of somatic cells were observed [47]. In addition, acetone can negatively affect ketosis and methane emissions in ruminants [48]. In the present study, the milk yield in the CON group was higher than that in the SCK group. In the SCK group, BHBA and acetoacetate were significantly higher (*p* < 0.05), and acetone tended to be higher (0.05 ≤ *p* < 0.1) than in the CON group. In addition, the synthesis and degradation of ketone body metabolic pathways differed significantly (*p* < 0.05). Klein et al. [14] reported that high concentrations of GPC are associated with low ketosis. In the present study, the sn-glycero-3-phosphocholine (GPC synonym) concentration was higher in the SCK group than in the CON group, but was not significant (*p* > 0.05), and the glycerophospholipid metabolism pathway showed a trend (0.05 ≤ *p* < 0.1).

Several studies on identifying metabolic markers of milk composition are being con-ducted to diagnose ketosis. Glycine in milk could be used as a marker for energy balance and metabolic status, and choline can supply methyl groups for cell growth in the mammary glands of lactating dairy cattle [18,49]. Xu et al. [18] reported that high concentrations of glycine and low concentrations of choline in milk are associated with negative energy balance in lactating dairy cattle. Sundekilde et al. [50] reported that carnitine, citrate, choline, and lactose are associated with milk coagulation properties. The coagulation parameters are positively affected by choline and negatively affected by carnitine, citrate, and lactose [50]. However, it is unclear how these metabolites are associated with milk coagulation processes [51]. In the present study, the lactose concentration in the CON group was higher than that in the SCK group, and glycine concentration in the SCK group was higher than that in the CON group, but the difference was not significant (*p* > 0.05). In addition, CON group choline tended to be higher (0.05 ≤ *p* < 0.1) compared with the SCK group. Somatic cell count (SCC) is associated with changes in protein, fat, and metabolites in milk [52]. Sundekile et al. [52] reported that when the SCC was higher in milk, acetate, butyrate, BHBA, isoleucine, and lactate concentrations were increased, while hippurate and fumarate concentrations were decreased. As acetate is associated with milk fat synthesis [53], the acetate concentration used in this study did not adversely affect milk composition. In the present study, the BHBA concentrations in the SCK group were significantly higher (*p* < 0.05) than that in the CON group. Acetate concentrations in the CON group were significantly higher (*p* < 0.05) than that in the SCK group. Among milk contents, riboflavin (vitamin B2) accounts for the second-highest ratio after calcium [54]; thus, it is thought to be a metabolite that can positively affect milk quality. In the present study, riboflavin tended to be higher (0.05 ≤ *p* < 0.1) in the CON group compared to the SCK group. Xanthine (purine metabolite) is catalyzed to uric acid (end product) by xanthine oxidase [55]. Xanthine oxidase has been isolated and purified from the milk fat globule membrane (MFGM) [56]. Studies have shown that MFGM has a protective effect against infectious diseases on the gut immune response and the gut microbiota [57,58,59]. In the present study, the xanthine concentration tended to be lower (0.05 ≤ *p* < 0.1) in the CON group than in the SCK group.

## 5. Conclusions

The metabolic profiles of the rumen fluid and milk samples from CON and SCK groups were obtained using ^1^H-NMR spectroscopy. In the rumen fluid, ruminant energy source metabolites including acetate, propionate, and glucose were higher in the CON group, whereas those metabolites associated with ketosis disease, including butyrate and BHBA were higher in the SCK group. In the milk, ketone body metabolites including BHBA, acetoacetate, and acetone were higher in the SCK group. Whereas, the varying results on milk quality as compared to previous studies and associated with milk quality metabolites including acetate and riboflavin were higher and xanthine was lower in the CON group. Future studies should focus on other metabolomics tools (LC-MS or GC-MS) and explore biomarkers related to ketosis.

## Figures and Tables

**Table 1:** Ingredients and nutrients of the experimental diets.

Items	Amount
Ingredients composition, % of DM	
Concentrate	15.30
Soybean meal	2.40
Corn silage	47.20
Alfalfa hay	7.10
Tall fescue	9.40
Timothy	5.90
Energy booster ^1^	7.10
Cash Gold ^1^	4.50
Lyzin-Plus ^2^	0.20
Limestone ^3^	0.20
Zin Care ^1^	0.10
Supex-F ^1^	0.50
Trace minerals ^4^	0.05
Vitamins premix ^5^	0.05
Chemical composition (% of DM basis)	
Dry matter (DM), %	53.2
CP	10.0
NDF	28.2
ADF	16.9
Ca	0.40
P	0.15

^1^ Cofavet, Cheonan, Republic of Korea. Zin Care, contained 16 GDU/g protease bromelain, 2.0 × 10^8^ cfu/g; Supex-F, contained 99% protected fat from palm oil. ^2^ A.N.Tech, Cheonan, Republic of Korea. Lyzin-Plus, contained 6.0% Zn, 0.9% Cu, 1.4% Mn, 5.0% chelated glycine. ^3^ Sungshin minefield, Jeongseon, Republic of Korea. ^4^ Trace minerals, contained 0.4% magnesium, 0.20% potassium, 4% sulfur, 0.08% sodium, 0.03% chlorine, 0.4 g of iron/kg, 60.042 g of zinc/kg, 16.125 g of copper/kg, and 42.375 g of manganese/kg. ^5^ Vitamins premix, provided approximately 5000 KIU of retinol/kg, 1000 KIU of cholecalciferol/kg, 33.5 g of tocopherol/kg, and 2.4 g of ascorbic acid/kg. CP, crude protein; NDF, neutral detergent fiber; ADF, acid detergent fiber; Ca, calcium; P, phosphorus.

**Table 2:** Summary of general information of metabolites in rumen fluid and milk in experimental Holstein cows.

General Information	Healthy	Subclinical Ketosis
*Rumen fluid*		
Detected metabolites (*n* ≥ 1)	145	171
Quantified metabolites (*n* = 3)	48	45
Classified chemical classes	13	13
Range of metabolites concentration	0.87~23,207.30 μM	7.30~22,139.17 μM
*Milk*		
Detected metabolites (*n* ≥ 1)	163	162
Quantified metabolites (*n* = 3)	69	84
Classified chemical classes	14	13
Range of metabolites concentration	1.00~98,861.57 μM	1.53~77,940.70 μM

**Table 3:** Differential enrichment of metabolites content of rumen fluid and milk between healthy and subclinical ketosis groups.

Metabolites	Classification	CON/SCK ^1^	*p*-Value	VIP Score ^2^	Fold Change ^3^
*Rumen Fluid*					
Butyrate	Organic acids	SCK	6.74 × 10^−4^	0.94	−0.14
*N*,*N*-dimethylformamide	Carboxylic acids	CON	1.15 × 10^−3^	2.17	0.78
Acetate	Organic acids	CON	3.06 × 10^−3^	0.72	0.09
Sucrose	Carbohydrates	SCK	1.18 × 10^−2^	1.81	−0.57
Glucose	Carbohydrates	CON	1.44 × 10^−2^	1.21	0.25
Propionate	Organic acids	CON	1.50 × 10^−2^	0.83	0.25
3-hydroxybutyrate	Lipids	SCK	2.20 × 10^−2^	1.87	−0.57
Maltose	Carbohydrates	SCK	3.36 × 10^−2^	1.51	−0.46
Valerate	Organic acids	SCK	3.83 × 10^−2^	0.81	−0.12
Methylamine	Amines	SCK	7.36 × 10^−2^	1.67	−0.44
Methionine	Amino acids	SCK	7.39 × 10^−2^	2.06	−0.82
Isopropanol	Alcohols	SCK	9.56 × 10^−2^	1.55	−0.64
*Milk*					
Galactitol	Carbohydrates	CON	1.81 × 10^−4^	1.92	0.68
1,3-dihydroxyacetone	Carbohydrates	CON	3.75 × 10^−4^	4.43	0.56
Maleate	Carboxylic acids	SCK	6.18 × 10^−4^	2.32	−1.05
γ-glutamylphenylalanine	Amino acids	CON	7.73 × 10^−4^	2.01	0.77
3-hydroxybutyrate	Lipids	SCK	5.28 × 10^−3^	1.87	−0.70
Acetoacetate	Carbohydrates	SCK	1.80 × 10^−2^	1.23	−0.32
5-aminolevulinate	Carboxylic acids	CON	2.11 × 10^−2^	1.91	0.78
Acetate	Organic acids	CON	2.98 × 10^−2^	1.53	0.46
Galactonate	Carbohydrates	SCK	4.15 × 10^−2^	0.92	−0.19
3-hydroxykynurenine	Organic acids	SCK	4.54 × 10^−2^	1.73	−0.54
Methylamine	Amines	CON	4.90 × 10^−2^	2.17	1.12
Acetone	Others	SCK	5.73 × 10^−2^	1.01	−0.25
Guanidoacetate	Carboxylic acids	SCK	7.68 × 10^−2^	1.01	−0.22
2-oxoisocaproate	Organic acids	SCK	7.94 × 10^−2^	1.56	−0.70
Xanthine	Nucleosides, Nucleotides	SCK	8.15 × 10^−2^	1.61	−0.77
Riboflavin	Others	CON	8.55 × 10^−2^	0.68	0.23
Choline	Lipids	CON	9.24 × 10^−2^	1.76	0.74
Trehalose	Carbohydrates	SCK	9.63 × 10^−2^	2.16	−2.00

^1^ CON/SCK, comparison between healthy (CON) and subclinical ketosis (SCK) group. ^2^ VIP Score, variable importance in the projection obtained from the partial least square-discriminant analysis model. ^3^ Fold Change, calculated as binary logarithm of the average concentration response ratio between CON and SCK group, where the positive value means that average concentration response of the metabolites in the former is larger than that in the latter and vice versa.

**Table 4:** Pathway analysis of significantly different rumen fluid and milk metabolites compared with healthy and subclinical ketosis groups.

Metabolic Pathway	Total Cmpd ^1^	Hits ^2^	*p*-Value	−Log (*p*-Value)	FDR ^3^	Impact ^4^
*Rumen fluid*						
Starch and sucrose metabolism	18	2	1.98 × 10^−4^	3.70	1.98 × 10^−3^	0.12
Pyruvate metabolism	22	1	3.07 × 10^−3^	2.51	1.02 × 10^−2^	0.06
Glyoxylate and dicarboxylate metabolism	32	1	3.07 × 10^−3^	2.51	1.02 × 10^−2^	0.00
Glycolysis and gluconeogenesis	26	2	8.43 × 10^−3^	2.07	1.69 × 10^−2^	0.03
Butanoate metabolism	15	2	1.04 × 10^−2^	1.98	1.69 × 10^−2^	0.00
Galactose metabolism	27	1	1.13 × 10^−2^	1.95	1.69 × 10^−2^	0.04
Propanoate metabolism	23	1	1.18 × 10^−2^	1.93	1.69 × 10^−2^	0.00
Synthesis and degradation of ketone bodies	5	1	2.01 × 10^−2^	1.70	2.51 × 10^−2^	0.00
Cysteine and methionine metabolism	33	1	7.77 × 10^−2^	1.11	7.77 × 10^−2^	0.10
Aminoacyl-tRNA biosynthesis	48	1	7.77 × 10^−2^	1.11	7.77 × 10^−2^	0.00
*Milk*						
Galactose metabolism	27	1	3.99 × 10^−4^	3.40	3.43 × 10^−3^	0.00
Glycerolipid metabolism	16	1	4.03 × 10^−4^	3.39	3.43 × 10^−3^	0.00
Glycine, serine and threonine metabolism	34	3	7.64 × 10^−4^	3.12	4.33 × 10^−3^	0.02
Synthesis and degradation of ketone bodies	5	2	3.24 × 10^−3^	2.49	1.10 × 10^−2^	0.60
Butanoate metabolism	15	2	3.24 × 10^−3^	2.49	1.10 × 10^−2^	0.11
Tyrosine metabolism	42	1	4.18 × 10^−3^	2.38	1.18 × 10^−2^	0.00
Arginine and proline metabolism	38	1	5.96 × 10^−3^	2.22	1.45 × 10^−2^	0.02
Valine, leucine and isoleucine degradation	40	2	1.01 × 10^−2^	2.00	2.14 × 10^−2^	0.01
Riboflavin metabolism	4	1	1.15 × 10^−2^	1.94	2.17 × 10^−2^	0.50
Pyruvate metabolism	22	1	4.27 × 10^−2^	1.37	5.56 × 10^−2^	0.06
Glycolysis and gluconeogenesis	26	1	4.27 × 10^−2^	1.37	5.56 × 10^−2^	0.03
Glyoxylate and dicarboxylate metabolism	32	1	4.27 × 10^−2^	1.37	5.56 × 10^−2^	0.00
Porphyrin and chlorophyll metabolism	30	1	4.36 × 10^−2^	1.36	5.56 × 10^−2^	0.03
Valine, leucine and isoleucine biosynthesis	8	1	4.58 × 10^−2^	1.34	5.56 × 10^−2^	0.00
Glycerophospholipid metabolism	36	1	5.82 × 10^−2^	1.23	6.60 × 10^−2^	0.03
Purine metabolism	66	1	6.23 × 10^−2^	1.21	6.62 × 10^−2^	0.03
Starch and sucrose metabolism	18	1	7.36 × 10^−2^	1.13	7.36 × 10^−2^	0.00

^1^ Total Cmpd, The total number of compounds in the pathway. ^2^ Hit, The actually matched number from the user uploaded data.^3^ FDR, The *p*-value adjusted false discovery rate. ^4^ Impact, The pathway impact value calculated from pathway topology analysis.

## Data Availability

The data presented in this study are available on request from the corresponding author.

## Supplementary Materials

The following are available online at https://www.mdpi.com/article/10.3390/ani11092526/s1, Tables S1 and S2: Quantified metabolites concentration in rumen fluid of healthy and subclinical ketosis groups by proton nuclear magnetic resonance spectroscopy analysis (Mean ± Standard deviation, *n* = 3), Tables S3 and S4: Quantified metabolites concentration in the milk of healthy and subclinical ketosis groups by proton nuclear magnetic resonance spectroscopy analysis (Mean ± Standard deviation, *n* = 3), Figure S1: The classification of detected rumen fluid metabolites according to chemical classes in healthy (a) and subclinical ketosis (b) groups by proton nuclear magnetic resonance spectroscopy analysis, Figure S2: The classification of detected milk metabolites according to chemical classes in healthy (a) and subclinical ketosis (b) groups by proton nuclear magnetic resonance spectroscopy analysis.
