# Supplementary material for: Metabolic Profiling of Rumen Fluid and Milk in Lactating Dairy Cattle Influenced by Subclinical Ketosis Using Proton Nuclear Magnetic Resonance Spectroscopy
Source: Animals (Basel). 2021 Aug 27;11(9):2526. doi: 10.3390/ani11092526 (PMC8471376; doi:10.3390/ani11092526)
Supplement: Supplementary file 1 [file animals-11-02526-s001.zip › animals-1256587-supplementary.pdf]

Supplementary Materials

Table S1. Quantified metabolites concentration in rumen fluid of healthy and subclinical ketosis groups by proton nuclear magnetic resonance spectroscopy analysis (Mean ± Standard deviation, n=3)

Metabolites (µM)	Healthy	Subclinical Ketosis	Metabolites (µM)	Healthy	Subclinical Ketosis
Alcohols			***Carboxylic acids***		
Isopropanol	16.07 ± 1.91	69.70 ± 68.87	2-hydroxyisobutyrate	2.97 ± 0.35	NQ
Methanol	2.40 ± 1.06	4.47 ± 1.65	3-hydroxyisovalerate	4.93 ± 4.04	4.93 ± 3.84
Amines			3-hydroxyphenylacetate	7.13 ± 3.69	11.27 ± 13.80
Histamine	1.43 ± 0.12	2.87 ± 2.45	Creatine phosphate	NQ	1.63 ± 0.90
Methylamine	Not quantified (NQ)	121.43 ± 23.66	Homovanillate	2.73 ± 3.28	1.97 ± 1.58
Amino acids			Malonate	2.40 ± 0.82	NQ
Anserine	4.83 ± 1.86	5.80 ± 6.93	*N*,*N*-dimethylformamide	0.73 ± 0.23	NQ
N-phenylacetylglycine	4.70 ± 0.98	4.60 ± 2.54	*N*-acetylglycine	14.67 ± 9.41	17.80 ± 5.21
π-methylhistidine	3.43 ± 0.40	6.43 ± 2.40	Pantothenate	4.30 ± 5.29	NQ
Methionine	NQ	8.40 ± 7.98	trans-aconitate	1.47 ± 0.31	NQ
Benzoic acids			***Imidazolinones***		
3,4-dihydroxybenzeneacetate	4.40 ± 1.51	NQ	Imidazole	NQ	10.00 ± 2.80
4-hydroxy-3-methoxymandelate	2.83 ± 2.82	NQ	***Indoles***		
4-hydroxyphenylacetate	NQ	4.87 ± 3.03	5-hydroxyindole-3-acetate	2.17 ± 1.44	2.77 ± 1.94
o-cresol	NQ	7.37 ± 2.54	***Lipids***		
p-cresol	NQ	7.67 ± 1.31	3-hydroxy-3-methylglutarate	19.30 ± 0.98	13.60 ± 10.31
Syringate	0.93 ± 0.40	0.67 ± 0.35	3-hydroxybutyrate	2.67 ± 1.84	9.50 ± 1.51
Carbohydrates			Caprate	48.43 ± 9.64	73.53 ± 20.53
1,3-dihydroxyacetone	NQ	3.27 ± 1.91	Choline	NQ	1.47 ± 0.93
Acetoacetate	4.37 ± 1.99	13.03 ± 10.93	Thymol	NQ	5.03 ± 2.21
Erythritol	7.00 ± 5.01	NQ	***Organic acids***		
Glucose	84.00 ± 10.85	46.17 ± 10.46	Acetate	16046.67 ± 892.85	12902.27 ± 903.23
Lactose	9.50 ± 3.61	12.97 ± 8.92	Butyrate	2626.10 ± 262.15	3569.03 ± 258.87
Maltose	11.77 ± 2.43	32.53 ± 14.75	Isobutyrate	161.10 ± 31.31	NQ
N-acetylglucosamine	9.90 ± 6.41	28.23 ± 22.55	Isovalerate	67.40 ± 51.61	74.83 ± 46.51
Pyruvate	11.93 ± 1.39	NQ	Phenylacetate	17.27 ± 8.52	27.80 ± 14.48
Succinate	4.67 ± 2.49	NQ	Propionate	4974.80 ± 104.04	3893.73 ± 629.24
Sucrose	1.43 ± 0.51	5.27 ± 2.21	Succinylacetone	7.07 ± 3.24	5.70 ± 5.48
			Valerate	288.70 ± 55.98	371.30 ± 34.54

Table S2. *Continued*

Metabolites (μM)	Healthy	Subclinical Ketosis
Others		
2-hydroxyphenylacetate	6.60 ± 2.09	6.03 ± 3.28
3-methylxanthine	0.57 ± 0.29	0.87 ± 0.21
3-phenylpropionate	110.37 ± 20.26	106.70 ± 23.72
4-pyridoxate	1.97 ± 0.85	2.13 ± 1.36
Acetone	5.00 ± 3.90	11.73 ± 3.96
Betaine	1.13 ± 0.90	6.53 ± 8.38
Biotin	8.10 ± 3.84	NQ
Dimethyl sulfone	3.07 ± 1.89	NQ
N-acetylserotonin	NQ	1.53 ± 0.45
Pyridoxine	0.70 ± 0.17	NQ

Table S3. Quantified metabolites concentration in milk of healthy and subclinical ketosis groups by proton nuclear magnetic resonance spectroscopy analysis (Mean ± Standard deviation, n=3)

Metabolites (µM)	Healthy	Subclinical Ketosis	Metabolites (µM)	Healthy	Subclinical Ketosis
Alcohols			***Carbohydrates***		
Methanol	6.23 ± 3.66	15.07 ± 6.77	1,3-dihydroxyacetone	4921.70 ± 515.73	NQ
Propylene glycol	Not quantified (NQ)	16.67 ± 16.64	Acetoacetate	35.27 ± 3.40	62.87 ± 7.77
Aliphatic acylic compounds			Fructose	182.97 ± 29.94	252.03 ± 93.93
O-phosphocholine	35.67 ± 43.74	168.63 ± 187.83	Galactitol	6481.13 ± 615.38	NQ
Trimethylamine *N*-oxide	24.67 ± 32.54	173.10 ± 292.72	Galactonate	45.50 ± 5.61	63.40 ± 24.19
Urea	285.43 ± 34.11	347.33 ± 84.61	Galactose	231.53 ± 72.30	111.77 ± 39.87
Amines			Glucitol	NQ	3908.50 ± 1199.66
Dimethylamine	NQ	12.17 ± 7.81	Isocitrate	NQ	172.83 ± 170.61
Histamine	5.00 ± 2.01	3.13 ± 0.45	Lactose	83056.53 ± 4999.75	61310.67 ± 2431.94
Methylamine	13.60 ± 11.99	NQ	Lactulose	NQ	142.90 ± 34.07
Sarcosine	9.87 ± 3.20	NQ	Maltose	51.97 ± 31.70	47.53 ± 3.76
Trimethylamine	0.80 ± 0.87	NQ	Mannose	156.90 ± 53.94	55.27 ± 77.78
Amino acids			*N*-acetylglucosamine	194.47 ± 46.44	208.67 ± 46.26
2-furoylglycine	NQ	2.53 ± 0.55	Sucrose	60.83 ± 13.60	48.73 ± 20.94
Anserine	20.53 ± 18.03	6.07 ± 1.65	Trehalose	NQ	37.57 ± 20.33
Creatine	94.07 ± 135.04	14.80 ± 6.03	Xylose	NQ	99.90 ± 55.04
3-glutamylphenylalanine	21.20 ± 6.08	NQ	***Carboxylic acids***		
Glycine	2139.00 ± 642.50	2169.40 ± 943.77	3-hydroxyphenylacetate	24.10 ± 6.55	16.70 ± 2.23
Methionine	6.90 ± 3.67	NQ	5-aminolevulinate	27.83 ± 15.85	3.90 ± 1.65
Valine	7.70 ± 7.79	4.27 ± 2.90	cis-aconitate	11.60 ± 3.76	5.30 ± 2.16
Benzoic acids			Creatine phosphate	184.37 ± 167.35	221.40 ± 54.66
3-hydroxymandelate	NQ	4.20 ± 0.79	Guanidoacetate	7051.37 ± 2822.75	10190.53 ± 1274.41
4-hydroxy-3-methoxymandelate	2.73 ± 1.33	3.70 ± 0.66	Homovanillate	2.33 ± 0.90	2.33 ± 0.21
4-hydroxyphenylacetate	NQ	5.73 ± 4.57	Maleate	NQ	1.77 ± 0.96
Acetylsalicylate	NQ	2.87 ± 1.37	Malonate	10.23 ± 4.14	13.47 ± 8.86
Isoeugenol	NQ	3.00 ± 2.77	*N,N*-dimethylformamide	4.80 ± 3.22	NQ
o-cresol	6.10 ± 6.34	NQ	*N*-acetylcysteine	NQ	20.43 ± 1.53
Salicylurate	NQ	3.80 ± 0.20	*N*-acetyltyrosine	6.40 ± 0.46	6.60 ± 2.55
Tartrate	60.77 ± 41.83	84.30 ± 8.58	*N*α-acetyllysine	NQ	9.97 ± 1.07
Vanillate	1.80 ± 0.36	1.73 ± 0.32	Pantothenate	NQ	6.43 ± 2.06

Table S4. *Continued*

Metabolites (µM)	Healthy	Subclinical Ketosis	Metabolites (µM)	Healthy	Subclinical Ketosis
Imidazolinones			***Others***		
Allantoin	17.17 ± 8.49	17.13 ± 4.12	1,3-dimethylurate	6.83 ± 5.05	8.17 ± 6.10
Creatinine	75.47 ± 22.09	68.80 ± 29.31	1,7-dimethylxanthine	NQ	4.77 ± 2.61
Imidazole	8.40 ± 6.07	7.60 ± 1.99	2-hydroxyphenylacetate	7.97 ± 1.74	NQ
Indoles			3-methylxanthine	1.30 ± 0.20	1.67 ± 1.07
5-hydroxyindole-3-acetate	1.97 ± 0.64	1.53 ± 0.15	4-pyridoxate	3.20 ± 3.16	1.63 ± 0.75
Lipids			Acetoin	NQ	38.70 ± 9.60
3-hydroxybutyrate	14.33 ± 3.61	59.70 ± 12.51	Acetone	33.97 ± 3.88	50.73 ± 8.60
Carnitine	22.77 ± 30.77	NQ	Arabinose	NQ	64.93 ± 30.56
Choline	247.63 ± 140.52	53.57 ± 38.58	Betaine	170.63 ± 204.89	201.77 ± 201.93
Ethylene glycol	NQ	3970.93 ± 940.15	Caffeine	3.37 ± 2.90	NQ
Glycolate	NQ	2071.60 ± 1193.08	Cellobiose	NQ	114.93 ± 34.43
Methylsuccinate	4.17 ± 0.98	NQ	Dimethyl sulfone	14.93 ± 1.31	11.33 ± 5.44
O-acetylcarnitine	92.90 ± 62.81	74.37 ± 41.80	Fucose	117.40 ± 52.40	NQ
Nucleosides, Nucleotides			Melatonin	NQ	2.23 ± 0.87
UDP-*N*-acetylglucosamine	1.57 ± 0.21	1.20 ± 0.26	*N*-acetylserotonin	1.93 ± 1.53	2.60 ± 1.15
Xanthine	NQ	11.50 ± 5.52	*N*-methylhydantoin	5.37 ± 5.12	1.87 ± 0.81
Organic acids			Pyridoxine	1.10 ± 0.17	1.43 ± 0.78
2-oxoisocaproate	NQ	1.00 ± 0.28	Riboflavin	1.47 ± 0.15	1.30 ± 0.10
3-hydroxykynurenine	NQ	5.87 ± 0.95	sn-glycero-3-phosphocholine	229.83 ± 125.46	305.43 ± 103.07
Acetate	43.80 ± 10.24	12.37 ± 4.71	Theophylline	3.50 ± 4.27	1.17 ± 0.50
Ferulate	1.37 ± 0.40	2.17 ± 1.69	τ-methylhistidine	NQ	3.47 ± 1.59
Formate	9.17 ± 1.90	6.40 ± 1.04			
Fumarate	7.10 ± 2.44	6.20 ± 1.91			
Gluconate	136.47 ± 98.22	106.83 ± 77.06			
N-nitrosodimethylamine	NQ	76.03 ± 101.45			
O-acetylcholine	52.83 ± 76.29	13.07 ± 5.86			

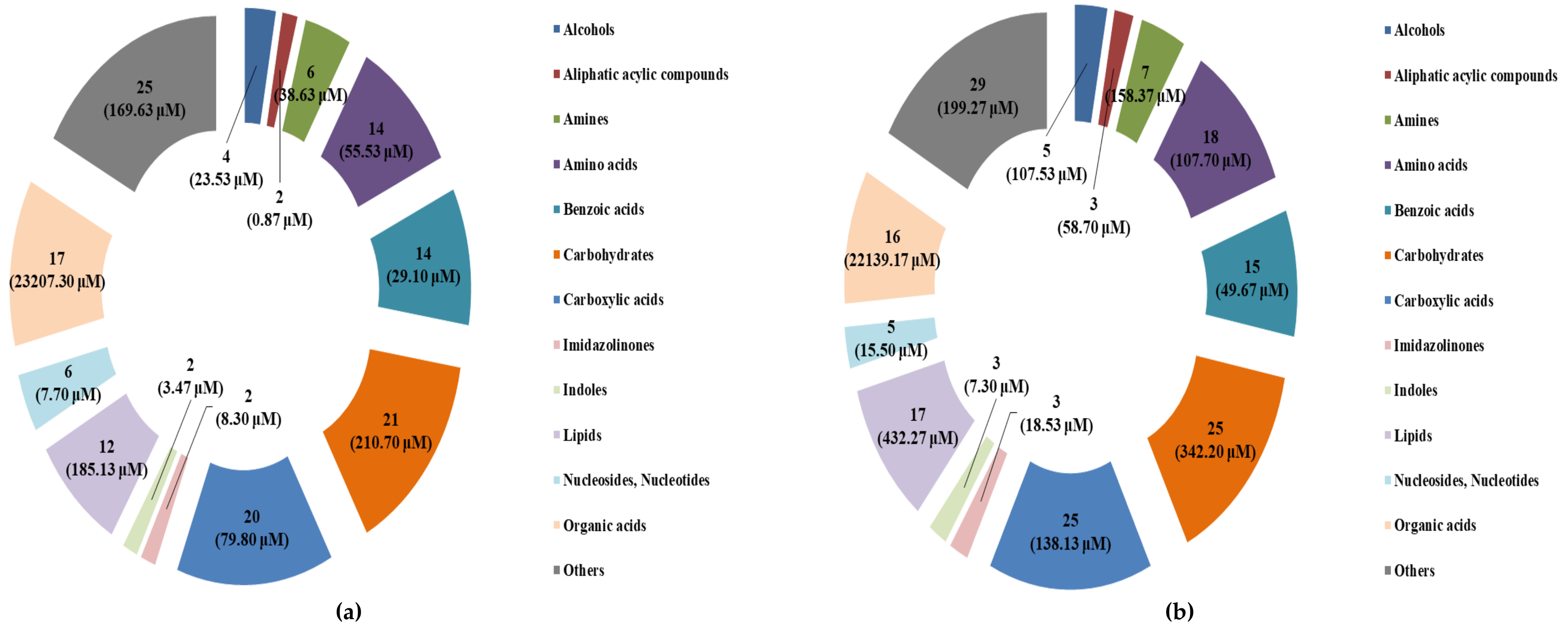

Figure S1. The classification of detected rumen fluid metabolites according to chemical classes in healthy (a) and subclinical ketosis (b) groups by proton nuclear magnetic resonance spectroscopy analysis.

Each square box color indicates the classification of metabolites, the number represents the detected metabolites, and the numbers in parentheses indicate the sum of the total concentration of the detected metabolites.

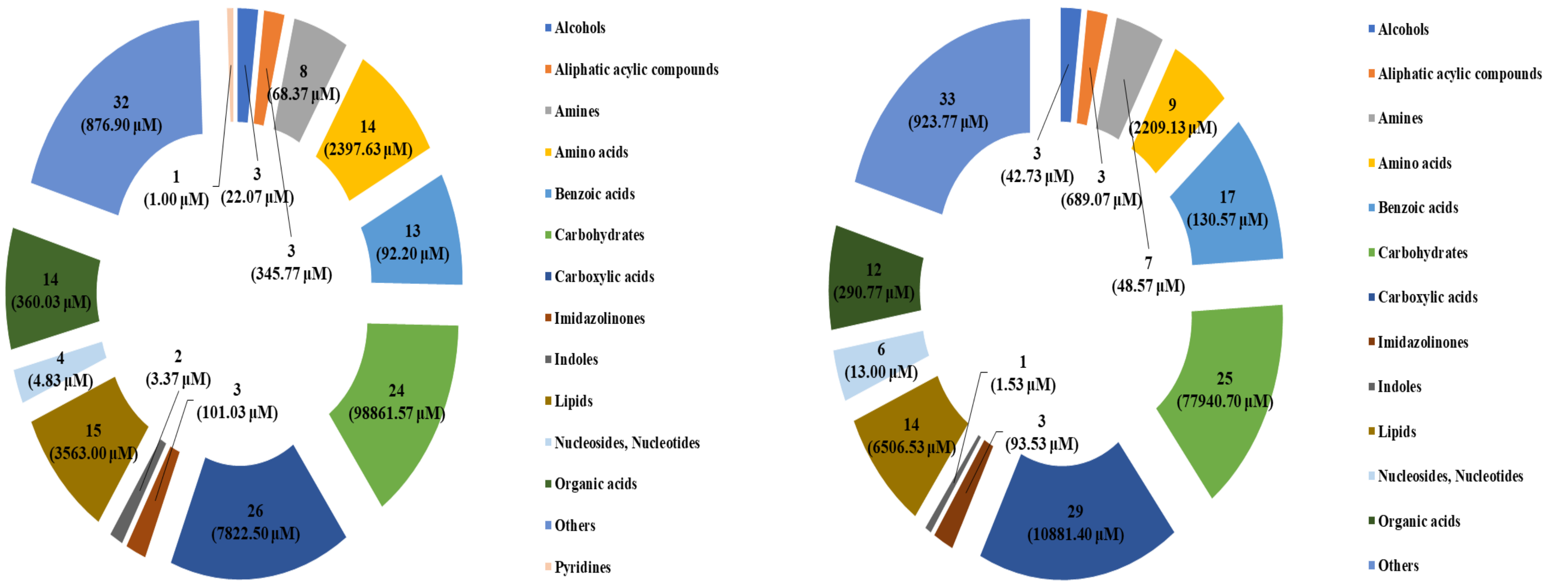

(a) **(b)**

Figure S2. The classification of detected milk metabolites according to chemical classes in healthy (a) and subclinical ketosis (b) groups by proton nuclear magnetic resonance spectroscopy analysis.

Each square box color indicates the classification of metabolites, the number represents the detected metabolites, and the numbers in parentheses indicate the sum of the total concentration of the detected metabolites.
